# On the Causes and Treatment of Tartar

**Published:** 1882-05

**Authors:** O. G. Duncan

**Affiliations:** England.


### ARTICLE IV.

### *On The Causes and Treatment of Tartar.*

BY O. G. DUNCAN, ENGLAND.

]Read before the Students' Society of the Dental Hospital of London.]

Mr. President and gentleman, the subject I have chosen for my paper this evening is one concerning which I found great difficulty in gathering information, so you must please excuse its brevity and its defects. It is also one that I fear will not leave room for much discussion; but perhaps the few ideas I have brought will be of interest to the new members of this Society, and if so the time will not have been spent in vain.

The deposit called "salivary calculus," or, to use the usual term, "tartar," is a collection of the earthy phosphates and other salts of the saliva, with epithelial scales and various extraneous matters entangled amongst them, found adhering to the various teeth.

A calculus is formed as a distinct concretion in one of the salivary glands or ducts; as a rule, when this is the case, like a calculus in the urinary bladder, there is a nucleus, which may be any foreign body that finds its way into the interior of the structure or organ. The two that I

have seen were each about the size of a pea, and quite soft. This was because there was no resisting surface against which they could be formed, and because from the inconvenience to which they gave rise they were not left long enough to become hardened. One of them was taken from the duct of a sublingual gland, which it so blocked up as to occasion an enormous accumulation of saliva. If the calculus can be felt by the finger it can, of course, be removed by a pair of forceps, but if it is some distance within the duct, so that it can only be felt with a probe, an incision must be made in order to reach it.

The formation of a calculous deposit upon the teeth, in a greater or less degree, may be said to be almost universal; for although in persons of good health it is possible by care to remove it by brushing soon after it is deposited (so that the teeth are kept nearly free from it,) still it is always being produced, and would be present if care were not used. We have seen that its source is the saliva, which is the secretion of the several salivary glands mixed with that of the small mucous glands; it is thin and watery, and contains a small quantity of animal matter, which is secreted by the sublingual gland and is called ptyalin. Ptyalin has a very peculiar property, it does not act upon proteid matters, not upon fats, but if mixed with starch and kept at a moderate temperature it converts the starch into dextrose or grape sugar, which is easily soluble, and useful for nutriment, whilst starch *per se* is quite insoluble, and therefore innutritious. The other constituents of saliva besides the ptyalin are the phosphates of lime and magnesia, the chloride of potassium, chloride of sodium (or common salt,) the carbonates of lime and magnesia, a small trace of sulphocyanate of potassium, and water. As regards the sulphocyanate of potassium it requires no great analysis to distinguish it; all that is necessary is to add a drop of the tincture of sesquichloride of iron to some saliva in a test tube, when you see the characteristic blood-red tinge develop itself. Now, the salts are only thrown down when the saliva is alkaline, as an acid reaction entirely prevents their precipitation. Thus we rarely see much, if any, around the upper incisors, because here the albuminous matter which is secreted from the mucous glands collects and ferments, and the resulting acidity serves to keep the salts in solution; whilst around the lower central incisors of the same patient we find them precipitated in abundance; this is because these teeth from their position are continually being bathed in alkaline saliva, so that no acidity can exist to keep them in solution.

Tartar collects in the largest quantity on the outer or buccal surface of the upper molars, just opposite the opening of Steno's duct—the duct leading from the parotid gland. This is more easily explained than in the case of the other situations where tartar collects, because the secretion from the parotid gland becomes turbid with crystals of carbonate of lime simply by exposure to the air, and this gland supplies more of the saliva than any other, as it is continually pouring out its secretion, whilst the sublingual and submaxillary glands only come into action at the thought of food or when required to act upon it when introduced into the mouth. The position also of the upper molars renders them less liable to friction, as they are protected by the cheek, whilst the deposit of tartar behind the lower incisors is greatly impeded by the continual action of the tongue.

Tartar exists in several varieties, which are named from variations in color and density. The usual color is whitish-yellow or buff, then we have the dark brown or black, and the green, but as far as I have tested them they do not differ chemically. The difference is due to the time occupied in deposition. If it is deposited quickly it will be of a light color and soft, whilst, on the contrary, if slowly it will be hard and dark. It is capable, also, of being stained by any coloring matter frequently taken into the mouth, as is the case of great tobacco smokers; or in patients who while taking any of the preperations of iron, drink tea just before or directly after, in which case the tartar will be stained perfectly black. The green variety, as a rule, is due to pus, which is formed by the irritation and inflammation caused by the tartar itself.

The way in which tartar is produced and the source from which it is derived have been stated and explained in various ways, but there is no doubt that that which we have noticed is the true explanation. That it is produced from the saliva is thoroughly proved by the fact that by chemical analysis the saliva is found to hold in solution the very elements of which the substance we are considering is composed, and that it is found exactly opposite the opening of the various ducts in the largest quantities, becoming thinner and thinner the further it is formed away from them.

With the exception of caries and violence this deposit is the most destructive influence to which the teeth can be exposed, but the effect it produces upon the teeth is only indirect, acting through the gums and alveoli, which it so destroys as to deprive the teeth of their support. If we look at a tooth so lost we see that the tartar reaches to nearly the apex of the fang, and the edge of this tartar nearest to where the margin of the gum was is very rough and sharp. The gum being in contact with this sharp rough margin become irritated and inflamed, the alveoli are absorbed, and the gums recede. As this process continues, fresh salts are precipitated on the exposed surfaces of the teeth, for tartar seems to have a greater affinity for the dental tissues than for itself.

A temporary support is produced by a large quantity of tartar, which very often joins two or three of the loosened teeth together. When this is the case they come away conjoined when it becomes necessary to extract any one of them.

We have all noticed that though the lower central incisors are less attacked by caries than any other teeth, they are the ones that are first lost by becoming loose, owing to this collection of tartar. Tartar used to be thought by some to be a preventive of caries. It was argued that where you see it in a large amount you see hardly any signs of caries, while, on the contrary, a person with a small amount of tartar, or perhaps none at all, is very susceptible to caries; this is due to the fact that tartar can only exist when the mouth is in a state unfavorable to caries, namely, in an alkaline condition.

As the accumulation of this substance affects not only the comfort and health of the mouth, but also the health of the whole system, the prevention of its deposition becomes of great importance, and there is no doubt that it may be obviated, or, at least, its immense accumulation lessened, by attending to the state of the stomach, and avoiding as much as possible any cause of irritation. But sometimes it is quite imposible to prevent its being deposited, as there are some people who are so susceptible to calculous deposits that no pains will stop its formation and it can only be kept down by constant attention, and by removing it while it is in a soft state. Very often when the tartar is of long duration the organic substances that are mixed with it decompose; this is the cause of the breath of these patients being so insufferable. Very often pus is seen oozing between the gums and teeth; this also makes the breath very offensive. If the patients rinse their mouths after each meal the collection of fats and other organic matter will be prevented, and thus, at any rate, they will keep their breath sweet. In advising patients as to the tooth brush after scaling, it is always best to tell them not to use one with hairs that are very stiff, as they are liable after the relief to scrub so hard, as if to make up for lost time, or so that it shall not occur again, that when you see them some time afterwards the necks of the teeth are very much worn away. I therefore tell them to have one with hairs not too closely set and very elastic, so as to remove the tartar, &c., from between the teeth.

I have found that most of the lotions which are sold to render "the teeth of pearly whiteness" contain dilute mineral acids and perfume. They certainly do make the teeth white and remove the tartar, but it is only at the cost of the enamel prisms, and lastly of the teeth themselves. Sometimes, also, patients will say they find vinegar cleans the teeth; this, of course, is a vegetable acid, and has a similar action to that of the mineral acids, only in a minor degree. The proper method, of course, is to remove the tartar with instruments adapted for the purpose, called scalers, and shaped for removing it from the different situations. There is no necessity for having them so large as some of the instruments are made. The best sets are Lord's and Howe's. The former's sickle scalers, as they are called from their shape, are very useful for removing the tartar from between the teeth. The scaling instruments should be highly tempered, and the edges kept sharp and clean. For the operation to be of benefit it must be performed thoroughly both on the external and internal surfaces as well as between the teeth, as the smallest particle left below the proper line of the gums will form a nucleus for further deposits, and thus keep the gums from recovering their normal position.

If the calculous deposit be present in such large quantities as to have loosened the teeth it is best to remove it at intervals of a few days, first removing that which is nearest to the apex of the fang. This will allow the gum to be again restored, and give the teeth a little chance of becoming tight; it will thus also permit of the remainder being removed without so much danger of removing the teeth with it. The tightening of them will be greatly assisted by frequently applying to them during the interval such astringents as tannic acid, alum, &c. After the tartar has been thoroughly removed it is a very good plan to nicely polish the teeth with pumice on a piece of cane, or for those who use the dental engine, with the wooden points sold for the purpose.—*British Journal of Dental Science.*

